# Bovine-Derived Xenografts Immobilized With Cryopreserved Stem Cells From Human Adipose and Dental Pulp Tissues Promote Bone Regeneration: A Radiographic and Histological Study

**DOI:** 10.3389/fbioe.2021.646690

**Published:** 2021-04-12

**Authors:** Yu Zhu, Shi-min Wei, Kai-xiao Yan, Ying-xin Gu, Hong-chang Lai, Shi-chong Qiao

**Affiliations:** ^1^Department of Implant Dentistry, Shanghai Ninth People’s Hospital, College of Stomatology, School of Medicine, Shanghai Jiao Tong University, Shanghai, China; ^2^National Clinical Research Center for Oral Diseases, Shanghai, China; ^3^Shanghai Key Laboratory of Stomatology, Shanghai Research Institute of Stomatology, Shanghai Jiao Tong University, Shanghai, China

**Keywords:** adipose tissue-derived stem cells, dental pulp stem cells, bone regeneration, calvarial defect, μCT examinations

## Abstract

Adipose tissue-derived stem cells (ADSCs) and dental pulp stem cells (DPSCs) have become promising sources for bone tissue engineering. Our study aimed at evaluating bone regeneration potential of cryopreserved ADSCs and DPSCs combined with bovine-derived xenografts with 10% porcine collagen. *In vitro* studies revealed that although DPSCs had higher proliferative abilities, ADSCs exhibited greater mineral depositions and higher osteogenic-related gene expression, indicating better osteogenic differentiation potential of ADSCs. After applying cryopreserved ADSCs and DPSCs in a critical-sized calvarial defect model, both cryopreserved mesenchymal stem cells significantly improved bone volume density and new bone area at 2, 4, and 8 weeks. Furthermore, the combined treatment with ADSCs and xenografts was more efficient in enhancing bone repair processes compared to combined treatment with DPCSs at all-time points. We also evaluated the sequential early bone healing process both histologically and radiographically, confirming a high agreement between these two methods. Based on these results, we propose grafting of the tissue-engineered construct seeded with cryopreserved ADSCs as a useful strategy in accelerating bone healing processes.

## Introduction

Bone defects caused by trauma, necrosis and tumors have long been recognized as a serious clinical problem due a high incidence of impaired healing (Rao and Stegemann, 2013). To date, mesenchymal stem cells (MSCs)-based therapy has emerged as an effective bone construction technique due to multi-differentiation potential of these cells (Nancarrow-Lei et al., 2017). Bone marrow-derived mesenchymal stem cells (BMSCs) are most commonly used for bone regeneration. However, the use of these MSCs is negatively affected by their inadequate numbers, invasive harvesting procedures and difficulties in controlling contamination (Xu et al., 2017). Recently, adipose tissue-derived stem cells (ADSCs) and dental pulp stem cells (DPSCs) have emerged as promising alternatives to BMSCs, as they can provide a relative abundance of MSCs (Caetano et al., 2019). The osteogenic potential of ADSCs has been investigated both *in vitro* and *in vivo*, and their efficiency have been evaluated in several small-scale clinical trials (Tajima et al., 2018). DPSCs, stem cells harvested from discarded extracted teeth, have shown easy recruitments with low invasiveness for patients and favorable self-renewal and differentiation potential (Kawashima, 2012; Pisciotta et al., 2012, 2020a,b; Lee et al., 2019). Moreover, recent studies have highlighted the peculiar embryological origin of DPSCs and provided further insights on their biological properties (Pisciotta et al., 2015a,b). During recent years, several studies have noted that subcutaneous implantation of ADSCs- and DPSCs-seeded scaffolds would result in ectopic bone formation, and both ADSCs and DPSCs are capable of improving critical-sized bone defect healing in different animal models (Shi et al., 2001; Lee et al., 2019; Novais et al., 2019). However, the isolation processes of ADSCs and DPSCs are intricate. Cryopreservation of MSCs would provide several benefits such as long-term storage, adjusting therapeutic cell doses and reducing contamination for safe clinical practices (Ma et al., 2012). Therefore, the cryopreserved storage and banking of MSCs would be a practical approach for MSCs-based therapies (Duchez et al., 2013). Nowadays, clinical application of cryopreservation has become an indispensable part of bone marrow and HSC transplantation (Seo et al., 2005; Davies et al., 2014). However, few studies have investigated the osteogenic capacities of cryopreserved DPSCs and ADSCs in bone healing processes.

A large panel of biomaterials, such as calcium phosphate cement (CPC), beta-tricalcium phosphate (β-TCP) and deproteinized bovine bone mineral (DBBM), has been utilized clinically for intra-bony defects reconstruction. β-TCP is a synthetic scaffold with rapid adsorptions and degradations, that can lead to insufficient bone formation during early healings (Hwang et al., 2012). CPC, a plastic paste, has low mechanical strengths and poor injectability (Matsumoto et al., 2014). Bio-Oss is a commercial DBBM with a structure similar to human cancellous bone. Due to its non-antigenic and favorable osteoconductive properties, Bio-Oss has been successfully used in bone regenerative therapies (Sanz-Martin et al., 2018). Bio-Oss Collagen is a composite of 90% deproteinized cancellous bone particles and 10% biodegradable collagen matrix of porcine origin. Collagen inside would facilitate graft handlings, resulting in better adaptation and stabilization (Nart et al., 2017). Animal experimental studies have shown that Bio-Oss Collagen was able to stimulate new bone formation locally (Wong and Rabie, 2010). Despite the evidence of Bio-Oss Collagen’s efficiency in bone repair, there are still only few studies that demonstrate (radiographically and histologically) consecutive changes in Bio-Oss Collagen within 2 months in a calvarial defect model. There is also limited evidence of the efficiency of combined applications of MSCs and Bio-Oss Collagen.

This study aimed to compare the osteogenic potential of Bio-Oss Collagen seeded with cryopreserved ADSCs and DPSCs. We examined the mineral deposition and osteogenic-related gene expression of both cell types *in vitro*. We also investigated the consecutive changes in MSCs-seeded scaffolds both radiographically and histologically in the calvarial defect model during early stages of bone repair. Our study would contribute to the application of tissue-engineered constructs in the treatment of bone defects.

## Materials and Methods

### Cell Culture

Adipose tissue-derived stem cells were isolated from human adipose tissues. DPSCs were isolated from human dental pulp. Procedures using human samples (adipose tissues and dental pulp) were conducted in accordance with the Declaration of Helsinki and approved by the Ethics Committee of Shanghai Ninth People’s Hospital affiliated to Shanghai Jiao Tong University (Protocol Number: SH9H-2019-TK34-1). Each patient signed the informed consent. Human adipose tissues were obtained from healthy patients who had undergone liposuction surgeries at Shanghai Ninth People’s Hospital. The primary ADSCs were obtained by enzymatic digestion as previously described (Zuk et al., 2001). Human dental pulp tissues were obtained from healthy human third molars extracted at Shanghai Ninth People’s Hospital. DPSCs were isolated and cultured as previously described (Gronthos et al., 2000). Both MSCs were cultured in Dulbecco’s modified Eagle’s medium (DMEM; Gibco, CA, United States) supplemented with 10% fetal bovine serum (FBS; Gibco). The medium was replenished every 3 days.

### Cell Cryopreservation

To prepare cryopreserved cells, passage 1 (P1) 80% confluent cultures were detached using 0.25% trypsin (Gibco). The supernatant was aspirated and equal volume of cells was resuspended in the 0.4% trypan blue solution for cell viability count. 1 × 10^6^ cells were then resuspended in cryogenic medium (90% FBS containing 10% dimethyl sulfoxide), and gradually frozen by incubating at 4°C for 1 h, 20°C for 2 h and 80°C overnight. Subsequently, frozen cell suspensions were transferred to liquid nitrogen storage. For cell recovery, ten vials of cryopreserved cells were thawed in an incubator at 37°C with 5% CO_2_ for 5 min. The content of each vial was then transferred into 15 mL tubes containing 5 mL cell medium and centrifuged at 900 *g* for 5 min to remove the residual cryogenic medium. Cell survival rate was measured immediately after thawing by using the trypan blue solution. Cells were then cultured to 80% confluence (approximately 24 h), and used for experimental procedures.

### Cell Morphological Analysis

Cryopreserved ADSCs and DPSCs were visualized by the light microscope (Olympus, Tokyo, Japan). Afterward, cells were fixed with 4% paraformaldehyde for 10 min and permeabilized with Triton X-100 (Amresco, OH, United States) for 5 min. Subsequently, cell actin was stained with rhodamine phalloidin (Sigma, MO, United States) for 30 min, nuclei were stained with DAPI (Sigma) for 5 min, and cell staining was analyzed using a fluorescent microscope (Nikon, Tokyo, Japan).

### Mesenchymal Markers Analysis

Cells were rinsed with phosphate buffered saline (PBS) and digested with 0.25% trypsin. After resuspending in the serum-containing medium, cell suspension was centrifuged and the supernatant discarded. Cell pellets were resuspended in PBS and incubated with the following fluorochrome-conjugated antibodies against CD34, CD45, CD73, CD90, CD105, and HLA-DR (BD, CA, United States) at 4°C for 30 min, protected from light (Lee et al., 2019; Pisciotta et al., 2020a). In order to assess the immune-phenotype and the stemness of the isolated cells, the expressions of surface markers were evaluated in ADSCs and DPSCs prior to cryopreservation. In order to verify if cryopreservation influences stemness of ADSCs and DPSCs, the surface marker profiles for ADSCs and DPSCs after thawing were evaluated again by using flow cytometry. Expression of mesenchymal markers was detected by the flow cytometry (BD, CA, United States).

### Cell Proliferation Assay

Cell proliferation rates were measured by cell counting kit-8 (CCK-8) assay. Briefly, cells were seeded at the density of 1 × 10^5^ cells/well. From day 1 to day 7, CCK-8 (Beyotime, Shanghai, China) was used according to manufacturer’s directions, and the absorbance at 450 nm was measured using a microplate reader (BioTek, VT, United States).

### Osteogenic Differentiation Assay

After reaching 70% confluence, cells were induced by replacing the growth medium with the osteogenic induction medium (a mixture of high-glucose DMEM, 10% FBS, 100 nM dexamethasone, 50 μM α-ascorbic acid, and 10 mM β-glycerophosphate). The osteogenic medium was replenished every 3 days.

At 1, 3, and 7 days, osteogenic differentiation was measured by alkaline phosphatase (ALP) staining. Briefly, cells were fixed with 4% paraformaldehyde and subsequently treated by a BCIP/NBTALP Color Development Kit (Beyotime). Samples were washed with distilled water and digital images were captured. The semiquantitative analysis of ALP activity was performed by evaluating the transformation of *p*-nitrophenyl-phosphate into p-nitrophenol (pNP). Briefly, cells were lysed with Triton X-100 and mixed with 100 μL *p*-nitrophenyl-phosphate substrate (1 mg/mL). Following the incubation in a 37°C water bath, the amount of pNP was measured by absorbance at 405 nm (BioTek). Total protein was quantified by the Pierce BCA Protein Assay Kit (Thermo, MA, United States). The ALP activity was calculated as absorbance (OD value) per milligram of total cellular protein.

At 7, 14, and 21 days, osteogenic differentiation was measured by alizarin red-S (ARS) staining. Briefly, cells were fixed with 4% paraformaldehyde and treated by 0.5% ARS staining solution (Sigma). Samples were washed with distilled water and digital images were captured. For the quantification analysis, stained samples were detached using 10% cetylpyridinium chloride (Sigma) and absorbance values at 590 nm were measured. Total protein was quantified by the Pierce BCA Protein Assay Kit (Thermo, MA, United States). The ARS was calculated as absorbance (OD value) per milligram of total cellular protein.

Expression levels of osteogenic-related genes were measured by the quantitative real-time polymerase chain reaction (qRT-PCR). Briefly, total RNA was extracted by using Trizol reagent (Invitrogen), RNA was reverse transcribed into cDNA using the PrimeScript 1st Strand cDNA Synthesis kit (Takara, Tokyo, Japan), and the gene expression was quantified using SYBR Premix Ex Taq II (Takara) on Bio-Rad qRT-PCR system. Each target gene was normalized to that of the housekeeping gene GAPDH and gene expression calculated based on the 2^–ΔΔ*CT*^ method. The △CT value were calculated according to the formula △CT = CT (gene of interest) – CT (GAPDH) in correlation analysis, and the 2^–△△*CT*^ was calculated according to the formula △△CT = △CT (experimental group) − ΔCT (control group) for determination of relative. Each experiment was repeated three times independently (Wang et al., 2016; Zhu et al., 2017).

The corresponding primers specific for osterix (OSX), osteopontin (OPN), osteocalcin (OCN), runt-related transcription factor 2 (RUNX2) and GAPDH are listed as follows, OSX: OSX-F, GCCAGAAGCTGTGAAACCTC, OSX-R, GCTG CAAGCTCTCCATAACC; OPN: OPN-F, GTACCCTGATGCTA CAGACG, OPN-R, TTCATAACTGTCCTTCCCAC; OCN: OCN-F, CACTCCTCGCCCTATTGGC, OCN-R, CCCTCCTGC TTGGACACAAAG; RUNX2: RUNX2-F, CTTCATTCGCCT CACAAACAAC, RUNX2-R, TCCTCCTGGAGAAAGTTTGCA; GAPDH: GAPDH-F, TTCACCACCATGGAGAAGGCT, GA PDH-R, TCTCATGGTTCACACCCATGA.

### Surgical Procedures

The calvarial bone defect model in nude mice was designed to evaluate bone repairs *in vivo*. Animals were obtained from the Animal Center. All the procedures involving animals followed the guidelines of the Animal Experimental Ethical Inspection of Shanghai Ninth People’s Hospital. For cell seedings, ADSCs and DPSCs were cultured in osteogenic medium for 7 days before being seeded on the Bio-Oss Collagen scaffold (2-mm diameter) at a density of 2 × 107 cells/mL. Thirty-six nude mice (6 weeks old) were randomized into four groups: Negative Control, Positive Control (Bio-Oss Collagen), Bio-Oss Collagen with ADSCs and Bio-Oss Collagen with DPSCs. All animal experiments were approved by the Animal Ethics Committee of Shanghai Ninth People’s Hospital affiliated to Shanghai Jiao Tong University (Protocol Number: SH9H-2019-A292-1). The production license and use license of the experimental animals were SCXK(SH)2018-0006 and SYXK(SH)2016-0016. The female BALB/c nude mice were fed in a specific pathogen free (SPF) environment. The surgical procedure has been described previously (Kustro et al., 2018). Briefly, nude mice were anesthetized by intramuscular injection of 50 mg/kg ketamine hydrochloride with 5 mg/kg diazepam under sterile conditions. The skin of cranium was incised and underlying tissues were detached. A 2-mm (diameter) calvarial defect was created in the middle of the parietal bone using the dental burr at 1,500 rpm. The negative control was left empty, and the positive control was grafted with Bio-Oss Collagen only. Two experimental groups were seeded either with ADSCs or with DPSCs. Tissues were then sutured by absorbable sutures. All nude mice were sacrificed at different time points and samples were fixed in 4% paraformaldehyde for further studies.

### Radiographic Analysis

Each sample was scanned by a μ-CT scan (Skyscan 1172; Bruker, Brussels Belgium) with a voltage of 50 kV, a current of 100 mA and a pixel resolution of 18 μm with a 0.5-mm aluminum filter. Three-dimensional (3D) image was reconstructed using CTAN image analysis software. Bone volume (BV), bone volume/total volume ratio (BV/TV) and trabecular number (Tb.N) within the volume of interest (VOI) were determined. Sagittal images of μ-CT examinations were then captured using DataViewer image analysis software. The two-dimensional (2D) μ-CT images of the target area were also visualized using pseudo-color maps (Figure 6A). The optimal threshold for discriminating between bone and grafts was determined. Tissues with a CT value between 700 and 2,000 Hounsfield units (Hu) were defined as new bone. Tissues with a CT value above 2,000 Hu were defined as Bio-Oss Collagen residuals. Based on these guidelines, the area of new bone and residual grafts were then calculated.

### Histologic Analysis

After μ-CT evaluation, samples were dehydrated in a series of graded alcohol concentrations and embedded in polymethylmethacrylate (PMMA). A diamond saw was used to cut initial sections which were further cut into the final slides of approximately 30 μm in thickness, stained with Van Gieson stain, and subjected to digital imaging. Markings of the various tissues were done with the Image-Pro Plus 6.0 software (Rockville, MD, United States). For the histomorphometric analysis, new bone areas were marked red, whereas bovine bone particles were marked green. Histological analysis of the groups was performed in a blinded manner, and the percentage of the new bone area relative to the entire calvarial defect area was then calculated.

After μ-CT evaluation, the bones were fixed in 10% paraformaldehyde and decalcified with 5% EDTA, followed by paraffin embedding. The sections from mid-defect regions were stained with hematoxylin and eosin (H&E). The sections were also deparaffined and rehydrated for immunohistochemical staining specific for osteocalcin. The antigen retrieval was performed by incubation with trypsin for 1 h at 37°C. The primary and secondary antibodies were rabbit anti-mouse osteocalcin (OCN) polyclonal Ab (1:200 dilution, Beyotime, China) and goat anti-rabbit, HRP-conjungated MAb (1:200 dilution), respectively. Finally, the samples were stained with Diaminobenzidine (DAB).

### Statistical Analysis

All experiments were performed in triplicates, and the data were expressed as means ± standard deviations. A one-way analysis of variation combined with a Student–Newman–Keuls *post hoc* test was used to determine the level of significance. The significance level was set at ^∗^*p* < 0.05 or ^∗∗^*p* < 0.01. The intra-observer reliability for the measurements of new bone percentage and bovine bone particles percentage by μ-CT were determined in terms of intraclass correlation coefficient (ICC). The agreement between radiographic and histological measurements of the new bone percentage and bovine bone particles percentage was determined by calculating the limits of agreement.

## Results

### Characterization of Cryopreserved Stem Cell

**FIGURE 1:**
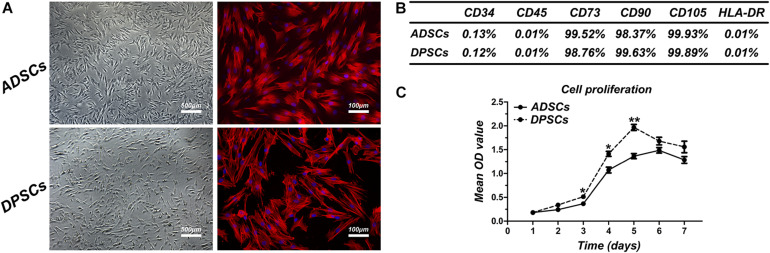
Comparison of cell characteristics of adipose-derived stem cells (ADSCs) and dental pulp stem cells (DPSCs). **(A)** Cell morphology of post-thawed ADSCs and DPSCs. **(B)** The results of flow cytometric analysis of cryopreserved ADSCs and DPSCs. **(C)** Proliferation curves of cryopreserved ADSCs and DPSCs. Data are presented as the mean ± standard deviation, *P < 0.05, **P < 0.01.

At P1, both ADSCs and DPSCs exhibited >95% viability before cryopreservation. Cryopreservation did not affect the number of viable cells for both cell types. The percentage of viable cells in post-thawed ADSC and DPSC was 93.58 ± 3.36% and 90.51 ± 2.86%, respectively (Table 1). Following cryopreservation, recovered ADSCs and DPSCs had spindle-shaped fibroblast-like morphology without cellular changes (Figure 1A). Flow cytometry analysis showed that both ADSCs and DPSCs expressed MSCs-associated surface markers, such as CD73, CD90, and CD105, but not CD34, CD45 and HLA-DR, confirming the immune-phenotype and the stemness itself of these isolated cells (Supplementary Figure 1). Moreover, the results revealed that both cell types after thawing were still positive for CD73, CD90, and CD105, but negative for CD34, CD45 and HLA-DR, demonstrating that cryostorage did not alter the expression of MSCs-associated markers in both cell lines (Figure 1B and Supplementary Figure 1). CCK-8 assay demonstrated that cryopreserved ADSCs and DPSCs displayed similar cell proliferative curves. However, significant difference was observed at 3, 4, and 5 days, suggesting that DPSCs had better cell proliferative capacities than ADSCs at an early stage (Figure 1C).

**TABLE 1:** Cell viability before cells were placed into cryostorage and immediately after thawing.

	**ADCSs**	**DPSCs**
Viability before cryostorage	96.34 ± 3.58	95.72 ± 4.01
Viability immediately after thawing	93.58 ± 3.36	90.51 ± 2.86

### Comparison of Osteogenic Potential

**FIGURE 2:**
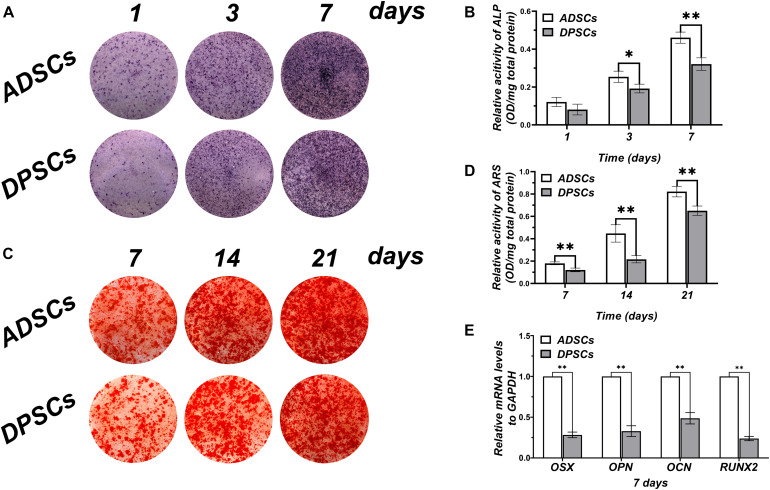
Comparison of osteogenic differentiation abilities of ADSCs and DPSCs. **(A)** Alkaline phosphate (ALP) staining at 1, 3, and 7 days after osteogenic induction. **(B)** ALP activity in both groups at each time-point determined by ALP semi-quantitatively. **(C)** Alizarin red S (ARS) staining at 7, 14, and 21 days after osteogenic induction. **(D)** Mineral deposition in both groups at each time-point determined by ARS semi-quantitatively. **(E)** The mRNA expression levels of osteogenic related genes in DPSCs and ADSCs at 7 day. Data are presented as the mean ± standard deviation, *P < 0.05, **P < 0.01.

Alkaline phosphatase staining was used to qualify mineral depositions at day 1, 3, and 7 (Figure 2A). No significant differences in mineral depositions were detected between ADSCs and DPSCs at day 1, whereas at day 3 and day 7 mineralization was markedly higher in ADSCs (Figure 2B). ARS staining of mineral depositions at day 7, 14, and 21 (Figure 2C) showed that ADSCs exhibited a more robust mineral capacity compared to DPSCs, with statistically significant differences at each time point (Figure 2D). After 7 days of culture, the osteogenic gene expressions of OSX, OPN, OCN, and RUNX2 were significantly higher in ADSCs than DPSCs (Figure 2E).

### Radiographic Evaluation of Calvarial Bone Healing

**FIGURE 3:**
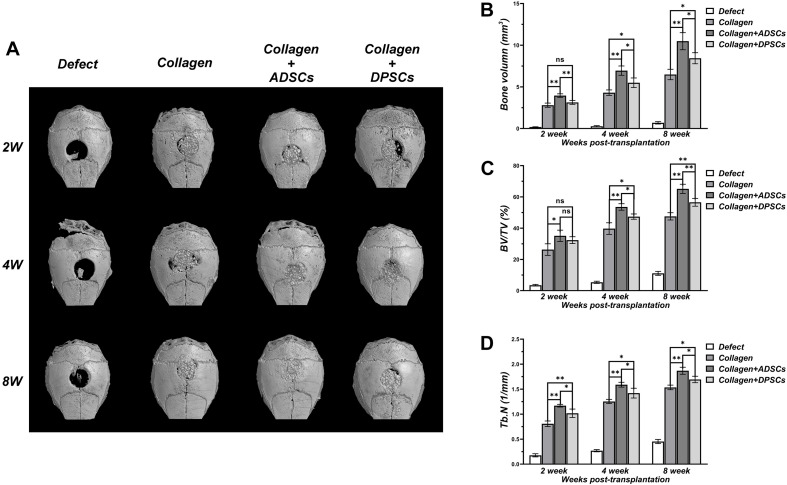
Three dimensional μCT analyses of bone regeneration in bone defect areas of Control, Bio-Oss Collagen, Bio-Oss Collagen + ADSCs, and Bio-Oss Collagen + DPSCs groups. **(A)** Representative μCT images at 2, 4, and 8 weeks. **(B)** Bone volume (BV), **(C)** Bone volume/total volume (BV/TV), **(D)** Trabecular number (Tb.N) by μCT at each time-point. Data are presented as the mean ± standard deviation, *P < 0.05, **P < 0.01.

As shown in Figure 3, there was a negligible bone healing in the negative control group by 8 weeks, indicating that a bone defect of 2 mm in diameter in the calvaria of nude mice failed to heal spontaneously. In Bio-Oss Collagen group, significant formation of bone islands from the bony edge around the original defect started only after 2 weeks, and ingrowth of new bones continued with time. In the Bio-Oss Collagen plus ADSCs and Bio-Oss Collagen plus DPSCs groups, new and continuous bone formation was observed as early as 2 weeks post-grafting, and the new bone nearly filled the entire defect by 8 weeks. Subsequent quantitative analyses confirmed that the negative control group had negligible increases in the BV (Figure 3B), BV/TV (Figure 3C), and Tb.N (Figure 3D). The BV, BV/TV, and Tb.N in samples only using Bio-Oss Collagen were low at 2 weeks and increased with time, reaching 6.49 ± 0.63 mm^3^, 47.56 ± 2.33%, 1.54 ± 0.05/mm, respectively, at 8 weeks (Figures 3B–D). The seeding of ADSCs accelerated the bone repair, with BV, BV/TV, and Tb.N reaching 3.96 ± 0.21 mm^3^, 35.13 ± 3.58% and 1.17 ± 0.03% (respectively) at 2 weeks, and 10.48 ± 1.03 mm^3^, 65.17 ± 2.96% and 1.87 ± 0.07% at 8 weeks. BV, BV/TV, and Tb.N were significantly higher in the ADSCs-seeded group than in the DPSCs-seeded or the positive control groups (Figures 3B–D).

**FIGURE 4:**
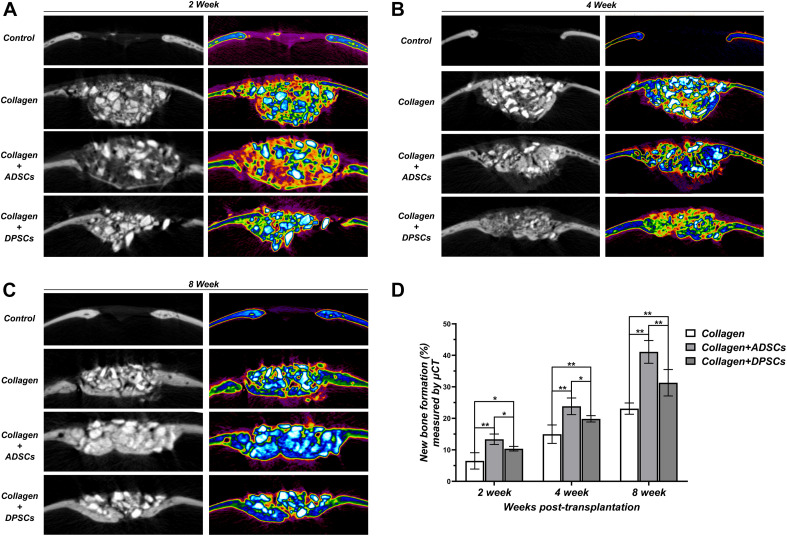
Sagittal images of μ-CT examinations in the region of interest (ROI) of Control, Bio-Oss Collagen, Bio-Oss Collagen + ADSCs, and Bio-Oss Collagen + DPSCs groups at **(A)** 2 weeks, **(B)** 4 weeks and **(C)** 8 weeks. Left panels: black-white tomograms. Right panels: images of equal-density pseudocolor. **(D)** Quantitative analysis of the new bone formation by using μ-CT examinations and pseudocolor processing. Data are presented as the mean ± standard deviation, *P < 0.05, **P < 0.01.

Based on the sagittal images captured by μ-CT, we hypothesized that MSCs-implanted scaffold would promote bone healing in bone defects. Here, we converted the black-white tomograms into images of equal-density pseudocolor by using pseudocolor processing, as illustrated in Figure 6A. Tissues with a CT value between 700 and 2,000 Hounsfield units (Hu) were defined as new bone. Tissues with a CT value above 2,000 Hu were defined as residual grafts. Bio-Oss Collagen transplanted with MSCs was able to regenerate the calvarial defect with a large amount of new bone-like structures compared to implantation with only Bio-Oss Collagen (Figures 4A–C). The amount of regenerated new bone at ADSCs-implanted sites was higher compared to that at the DPSCs-implanted site (Figures 4A–C). Based on pseudocolor images and subsequent data analysis, the seeding of MSCs was capable of promoting new bone formation in the calvarial defects at 2, 4, and 8 weeks (Figure 4D). Moreover, larger amounts of new bone formation were detected in ADSCs-seeded sites than in DPSCs-seeded sites. The new bone formation of ADSCs-seeded group reached 13.36 ± 1.67%, 23.83 ± 2.65%, and 41.09 ± 3.62% at 2, 4, and 8 weeks respectively, and that of DPSCs-seeded group reached 10.36 ± 0.73%, 19.85 ± 1.01%, and 31.31 ± 4.19%, respectively (Figure 4D).

### Histologic Evaluation of Calvarial Bone Healing

**FIGURE 5:**
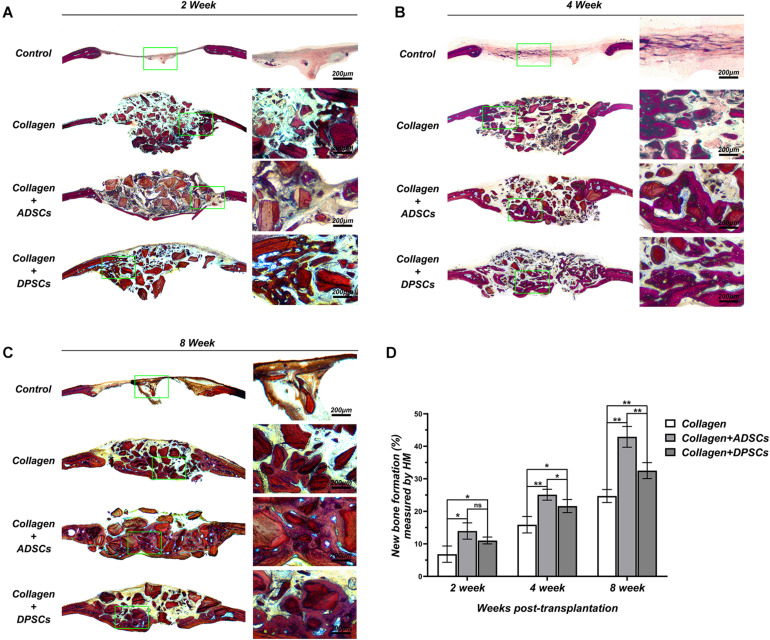
Histological slices in the sagittal plane in the region of interest (ROI) of Control, Bio-Oss Collagen, Bio-Oss Collagen + ADSCs, and Bio-Oss Collagen + DPSCs groups at **(A)** 2 weeks, **(B)** 4 weeks, and **(C)** 8 weeks. Right panels: images of green-bowed area in left panels. **(D)** Quantitative analysis of the new bone formation by using histological measurement. Data are presented as the mean ± standard deviation, *P < 0.05, **P < 0.01.

Figures 5A–C shows representative histological sections of four groups at 2, 4, and 8 weeks. No signs of inflammation or infection, induced by grafting materials or MSCs, were detected. Based on the Van Gieson staining, the bone repair was more noticeable on the surface of the bony edge around the original defect as well as from the periosteum on the parietal bone. In the negative control group, no bony tissue was observed and only connective tissue formation was evident at 2 weeks. After 6 weeks of healing, only mild new bone formation was observed at the defect margins. In the positive control group, graft particles were merely surrounded by dense connective tissue at 2 weeks. After 6 weeks, direct contact was observed between the new bone and the residual particles, indicating the osteoconductivity of Bio-Oss Collagen (Figures 5A–C). The groups seeded with ADSCs or DPSCs both showed a higher new bone formation in the bone defect area than the Bio-Oss Collagen only group. At 4 and 8 weeks, new bone grew around the graft particles and visibly infiltrated the porous structure. The degradation of the xenografts provided the space for subsequent new bone formation, without causing its structure to collapse. Here, Both MSCs-seeded groups not only induced horizontal bone defect closure, but also maintained the new bone height. Larger extent of new bone formation has been observed in ADSCs-seeded group than in DPSCs-seeded group (Figures 5A–C). The percentage of new bone formation in groups where Bio-Oss Collagen was used was significantly higher than that in the negative control group at 2, 4, and 8 weeks (Figure 5D). Moreover, a larger extent of new bone formation was estimated in ADSCs-seeded groups than in DPSCs-seeded groups. Quantitative analysis of the new bone formation by histological measurement showed that ADSCs-seeded group reached 13.95 ± 2.50%, 25.11 ± 1.67%, and 42.90 ± 3.19% of new bone formation at 2, 4, and 8 weeks, while DPSCs-seeded group reached 11.04 ± 1.05%, 21.62 ± 2.01%, and 32.51 ± 2.46%, respectively (Figure 5D). In general, these results indicated that the osteoconductive capacity of Bio-Oss Collagen was improved by the addition of ADSCs or DPSCs.

The H&E staining of tissue sections (Supplementary Figure 2a) further confirmed that formation of bone matrix was evident in MSCs-seeded groups, yet the new bone was randomly aligned and fibrous tissues were still evident. Moreover, the ADSCs-seeded group gave rise to superior formation of new bones in comparison to the DPSCs-seeded group (Supplementary Figure 2b). Immunohistochemical staining (Supplementary Figure 2c) further showed that OCN was abundantly deposited in MSCs-seeded groups but was relatively poorly distributed in the Bio-Oss Collagen only group. Quantitative results further showed that the deposition of OCN was lowest in the Collagen only group, indicating the lack of remodeling (Supplementary Figure 2d). Supplementary Figure 2 altogether demonstrated that the use of ADSCs and DPSCs significantly ameliorated the healing of calvarial bone defects thanks to active bone regeneration and remodeling.

### Comparison Between Histomorphometry and μCT

The intra-observer reliability for the percentages of new bone and graft materials measured by μCT is summarized in Table 2. The mean changes in scores between two consecutive measurements were small. Calculating the reliability of measurements of new bone and residual materials by μCT resulted in ICCs of 0.97 and 0.91, respectively. These results indicated high intra-observer reliability.

**TABLE 2:** The inter-observer reliability for the measurements of new bone percentage (%) and residual materials percentage (%) by μCT.

	**Difference between means**	**Intraclass correlation coefficient**
New Bone percentage (%) measured by μCT	1.907	0.97
Residual Materials percentage (%) measured by μCT	0.901	0.91

**FIGURE 6:**
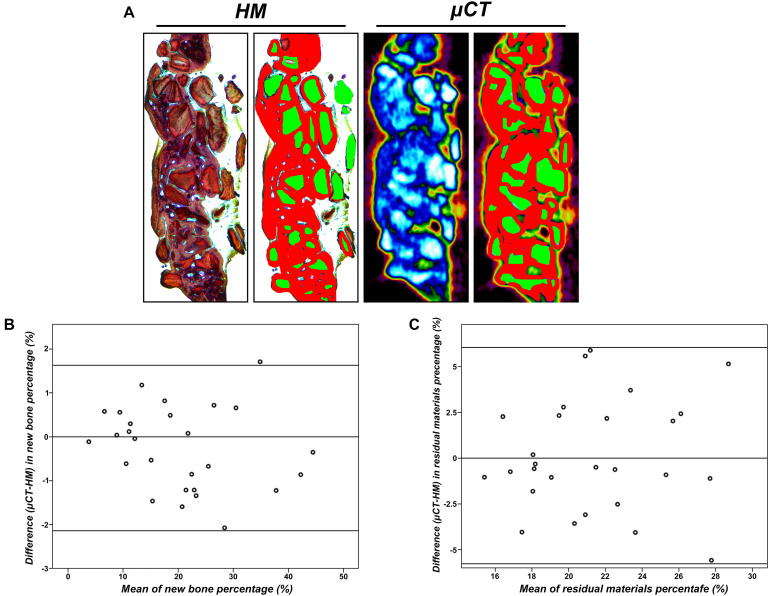
**(A)** View as used for radiographical and histomorphometrical analyses in the μ-CT or histological slices. Red, newly formed bone; green, residual particles (×10). **(B)** Agreement between μCT and histomorphometry (HM) for the measurement of new bone percentage (%). **(C)** Agreement between μCT and HM for the measurement of residual particle percentage (%).

As shown in Figures 6B,C, we compared the histomorphometry (HM) and μ-CT for an evaluation of new bone formation and residual scaffolds. The high agreement between HM and μ-CT indicated that the boundary of bone/no bone applied to the HM and μ-CT 2D data set was apparently well chosen. Here, the 95% confidence interval between the limits of agreement was larger in residual material measurements compared to new bone formation measurements, indicating more agreement for the measurement of the new bone area. Calibration of μ-CT for new bone remained difficult due to modeling and incorporation, and μ-CT appeared to underestimate new bone, especially in early bone healings.

## Discussion

With easy accessibility, high proliferation and differentiation capacities, ADSCs and DPSCs have become promising sources for bone regeneration (Pisciotta et al., 2012, 2020a; Xu et al., 2019). In the current study, we focused on the osteogenic differentiation of ADSCs and DPSCs. However, there is no consensus yet on the comparative efficiency of these two cell types. While some studies indicated that the clinical application of ADSCs was as effective or more effective than DPSCs, other reports concluded that DPSCs were superior to ADSCs (Jin et al., 2019; Kim et al., 2019; Terunuma et al., 2019). This discrepancy may be due to the different culture or induction setup as well as the different source of the cells. Another possible explanation is that MSCs, in particular DPSCs, might be a heterogeneous population of cells (Pisciotta et al., 2020a). Here, our data demonstrated that osteogenic factors (a mixture of dexamethasone, β-glycerophosphate and α-ascorbic acid) were able to efficiently induce osteogenic differentiation of ADSCs and DPSCs, suggesting that both cell types have osteogenic potential. Following cryopreservation, recovered ADSCs and DPSCs exhibited a normal fibroblast-like morphology, and the proportion of viable cells was not affected by cryopreservation. Based on the hypothesis that more than a single stem cell population might reside within the dental pulp (Pisciotta et al., 2015b), immunophenotypical characterization of isolated cells should be defined at the proper time. Here, ADSCs and DPSCs were positive for CD73, CD90, and CD105, but negative for CD34, CD45 and HLA-DR, confirming the immune-phenotype and the stemness itself of these isolated cells. Moreover, the surface marker expression profiles of ADSCs and DPSCs were not altered after thawing, demonstrating that the stemness were not influenced by the process of cryopreservation. Together, these results suggest that cryopreserved ADSCs and DPSCs may be a good source of MSCs for future translational research. However, viability of thawed stem cells appears to be cell-type specific, as viabilities of ADSCs and DPSCs immediately following post-thaw culture were 93.58 and 90.51%, respectively. These findings are of great interest, as recent studies showed that a population of multilineage differentiating stress-enduring cells in adipose tissue was able to endure extreme stresses such as hypoxia, serum deprivation, low temperatures and exposure to proteolytic enzymes (Ma et al., 2012). Although DPSCs had higher proliferative ability than ADSCs following cryostorage, *in vitro* experiments have shown higher ALP activities, more obvious mineral depositions and stronger osteogenic gene expression in the ADSCs-seeded group. These results suggest that the osteogenic differentiation potential of ADSCs was superior to that of DPSCs. The underlying mechanism of this effect remains unclear.

In order to properly evaluate bone healing ability of tissue-engineered constructs, a critical-size defect (CSD) must be large enough to create a non-viable space that inhibits indirect bone healing to bridge the defect spontaneously (Liao et al., 2014). In our study, a bone defect of 2 mm in diameter was created on the calvaria of nude mice. There was minimal morbidity due to this procedure as most of the mice were in good health and condition after the surgery. In our study, the defect in the control group remained unrepaired by 8 weeks, becoming non-union with fibrous connective tissue in the areas in which bone formation was absent, thereby confirming the creation of the CSD. Early works have shown that bone wounds will be filled at first with well vascularized connective tissue, followed by immature bone replacement, which will, in turn, be remodeled into mature bone (Saha et al., 2019). In our study, collective analyses of radiographic and histological data revealed that the calvarial defects filled with Bio-Oss Collagen had attained increased bone volume and more new bone area compared with unfilled defects. At 8 weeks post-surgery, the bone in treated groups appeared to have undergone significant anatomical remodeling and was returning to an intact physiological bonelike morphology. Based on the previous results, MSCs transplantation to regenerate bone showed good potential over other current treatment options (Jin et al., 2019). Given that transplantation of ADSCs and BMSCs revealed good results (in terms of higher bone volume and larger new bone area) in repairing the artificially created bone defect, we hypothesized that the addition of MSCs (at least in the form of ADSCs and DPSCs) would accelerate the bone healing process. We did not track the fate of the ADSCs and DPSCs used in our experiments *in vivo*. However, past reports demonstrated persistent engraftment of human MSCs when transplanted into immunoincompetent xenogenic models (Liao et al., 2014). We hypothesize therefore, that our MSCs transplanted into the nude mice calvarias might survive the initial transplantation and engraft within the defect. Among all evaluated groups in our study, samples seeded with ADSCs appeared to be the closest to being completely restructured after 8 weeks. The advantage of using ADSCs for bone regeneration was also confirmed by quantitative results both radiographically and histologically. Based on these results, we suggest that ADSCs are a promising source of stem cells in bone tissue engineering. However, it remains unclear how ADSCs and DPSCs contributed to the bone regeneration.

A local microenvironment generated by interactions among scaffolds, stem cells and native cells may interfere with osteogenic activity. Therefore, further research is required to seek the optimal conditions for MSCs to induce bone regeneration (Mokbel et al., 2008). Bio-Oss Collagen was specifically chosen as the carrier for the MSCs, since it is a biomaterial of natural origin that consists of bovine cancellous bone mineral particles and purified porcine collagen fibers (Wang et al., 2019). Our results indicated that Bio-Oss Collagen may support the growth and osteogenic differentiation of ADSCs and DPSCs, indicating that Bio-Oss Collagen was a good candidate material for bone tissue engineering. In our model, the defect passed through the calvarial bone, forcing the material to undergo intracranial pressure from the side of the dura mater. Such pressure could simulate the mucosal tissue shrinkage and compressive pressure of Schneider’s membrane (Lohmann et al., 2017). The Bio-Oss Collagen gave structural support and blocked the surrounding soft tissues from bulging into the defect. Thus, a bone defect treated with this composite would not require additional fixation to restrain the defect site. Additionally, we showed that Bio-Oss Collagen led to vertical bone augmentation in the calvarium of mice. In our study, the increase in the newly formed bone in the region of interest from 2 to 8 weeks indicated progressive bone regeneration. Histological analysis also demonstrated that Bio-Oss Collagen had a significant local osteogenic effect. It is possible that the surfaces of the composite facilitated mineralization and provided a source of calcium ions, further supporting mineralization. Interestingly, the residual particles were merely surrounded but not absorbed by the newly formed tissue. We inferred that the degradation of collagen fibers might provide the space for cellular invasion and subsequent bone tissue growth within the composite, without causing its structure to collapse. Further research is still needed to gain further understanding on the bone forming mechanism of Bio-Oss Collagen constructs and to optimize their use.

While histomorphometry is reliable when assessing bone healings, it is restricted by material losses during manufacturing and possible preparation artifacts (Gielkens et al., 2008). By contrast, μ-CT offers several advantages over histomorphometry, such as multiple analysis options, fast calculation and lower number of animals required (Kochi et al., 2010). While conventional micro-CT measurements cannot distinguish bone substitutes and new bone formation, the modern μ-CT scans provide higher spatial and temporal resolution imaging to capture detailed anatomical images. Furthermore, image conversion with pseudo color using new analyzing software, allows the display of gray values and the visualization of new bone borders. In the current study, we compared new bone areas on μ-CT slices with those on histological sections and found a high correlation and good accuracy between these two methods, verifying previous reports (He et al., 2017). The high agreement between μ-CT and HM also indicated that the boundary of bone/no bone applied to μ-CT slices was apparently well chosen. Our study, therefore, may be considered as a pilot study to evaluate the suitability of μ-CT for quantitative measurement of new bone formation. A limitation of our study is that the overall statement regarding μ-CT measurements may be influenced by biological variances as well as possible errors of the methodology. Complying with the principles of animal testing, a limited number of animals was chosen, with a large deviation within the groups. Our results indicated a slightly better bone regeneration detected by histological measurement compared to μ-CT scans. The reason for this discrepancy might be the failure to detect immature bone tissue with μ-CT scans. Future experiments are needed to further minimize potential artifacts and maximize bone contrast in both experimental and clinical research.

Cryopreserved MSCs, which could circumvent the preparation process before transplantation and provide standardized material on demand, may represent a novel bone regenerative cell therapy (Motoike et al., 2018). However, to date few studies have investigated the regenerative properties of both cryopreserved ADSCs and DPSCs on synthetic scaffolds for therapeutic use (Papaccio et al., 2006). In general, MSCs might be damaged during the cooling and defrosting procedures (Kojima et al., 2015). When MSCs are frozen, a cluster of intracellular ice crystals grows and injures the cellular membranes (Shikata et al., 2016). In our study, we found that cryopreservation did not affect the number of viable cells and the expression of MSCs-associated surface markers for both cell types, indicating that ADSCs and DPSCs were robust enough to survive and remain functional when subjected to particular cryopreservation protocols. Here, the cryoprotectant DMSO was used to achieve proper vitrification and succeeded in long-term cryopreservation. For MSCs cryopreservation, we also found that transplantation of cryopreserved ADSCs and DPSCs induced bone regeneration up to 8 weeks in a calvarial defect model. The overall results of this study encouraged the application of cryopreserved ADSCs and DPSCs in bone healing process, since successful cryopreservation of MSCs can omit the preparation process and enable a reliable supply of quality controlled material as needed. However, an increased understanding of the underlying molecular mechanisms involved in the bone repaired process is still needed to expand the applications of cryopreserved MSCs.

In summary, we used tissue-engineered constructs for the treatment of a critical-sized calvarial defect. Interestingly, combined treatment of Bio-Oss Collagen with cryopreserved MSCs enhanced the bone healing process, suggesting that the use of cryopreserved ADSCs and DPSCs might be a promising strategy, especially for the treatment of compound fractures and large bone defects. We have further demonstrated that compared with cryopreserved DPSCs, cryopreserved ADSCs exhibited enhanced mineralization capability, and increased expression of osteogenic-related genes. *In vivo* experiments have also shown that cryopreserved ADSCs have stronger bone repair ability. Taken together, our results indicate that cryopreserved ADSCs are more suitable as seed cells in bone engineering than cryopreserved DPSCs. Our study also provided evidence that ADSCs and DPSCs can be easily cryopreserved and recovered. This rendered them a potentially useful and reliable source of MSCs for delayed therapies, designed for bone repairs upon patients’ needs. There were limitations to our study. The number of animals was insufficient due to ethical restrictions. Another limitation was that the present study did not provide evidence of long-term effects. Furthermore, because the healing calvaria was exposed to weak mechanical stimuli, experiments need to be repeated in craniofacial bones submitted to stronger constraints. In addition, we failed to track the seeded stem cells within the implant site, which need to be further explored to get more solid evidences concerning the contribution of human stem cells.

## Data Availability Statement

The authors acknowledge that the data presented in this study must be deposited and made publicly available in an acceptable repository, prior to publication. Frontiers cannot accept a manuscript that does not adhere to our open data policies.

## Ethics Statement

The studies involving human participants were reviewed and approved by Ethics Committee of Shanghai Ninth People’s Hospital affiliated to Shanghai Jiao Tong University (Protocol Number: SH9H-2019-TK34-1). The patients/participants provided their written informed consent to participate in this study. The animal study was reviewed and approved by the Animal Ethics Committee of Shanghai Ninth People’s Hospital affiliated to Shanghai Jiao Tong University (Protocol Number: SH9H-2019-A292-1) and followed the guidelines of the Animal Experimental Ethical Inspection of Shanghai Ninth People’s Hospital.

## Author Contributions

YZ and S-mW conceived and designed the study, and wrote the manuscript. K-xY and Y-xG were involved in literature search and data collection, and analyzed the data. H-cL and S-cQ reviewed and edited the manuscript. All authors read and approved the final manuscript.

## Conflict of Interest

The authors declare that the research was conducted in the absence of any commercial or financial relationships that could be construed as a potential conflict of interest.

## Supplementary Material

Supplementary Figure 1 Characterization of surface marker profiles for ADSCs and DPSCs prior to and after cryopreservation. The flow cytometry results revealed that the ADSCs and DPSCs were always positive for CD73, CD90, and CD105, but negative for CD34, CD45, and HLA-DR.

Supplementary Figure 2 Calvarial bone repair at 8 weeks in Control, Bio-Oss Collagen, Bio-Oss Collagen + ADSCs and Bio-Oss Collagen + DPSCs groups. **(a)** Hematoxylin and eosin (H&E) stained images. **(b)** New bone area percentages in the defect region as assessed by histomorphometric analyses. **(c)** Immunohistochemical staining specific for osteocalcin (OCN). **(d)** OCN immunostaining percentages as assessed by DAB total area/tissue total area analyses. Data are presented as the mean ± standard deviation, ^∗^*P* < 0.05, ^∗∗^*P* < 0.01.
