# Supplementary figures and images for: Bovine-Derived Xenografts Immobilized With Cryopreserved Stem Cells From Human Adipose and Dental Pulp Tissues Promote Bone Regeneration: A Radiographic and Histological Study
Source: Front Bioeng Biotechnol. 2021 Apr 12;9:646690. doi: 10.3389/fbioe.2021.646690 (PMC8075412; doi:10.3389/fbioe.2021.646690)

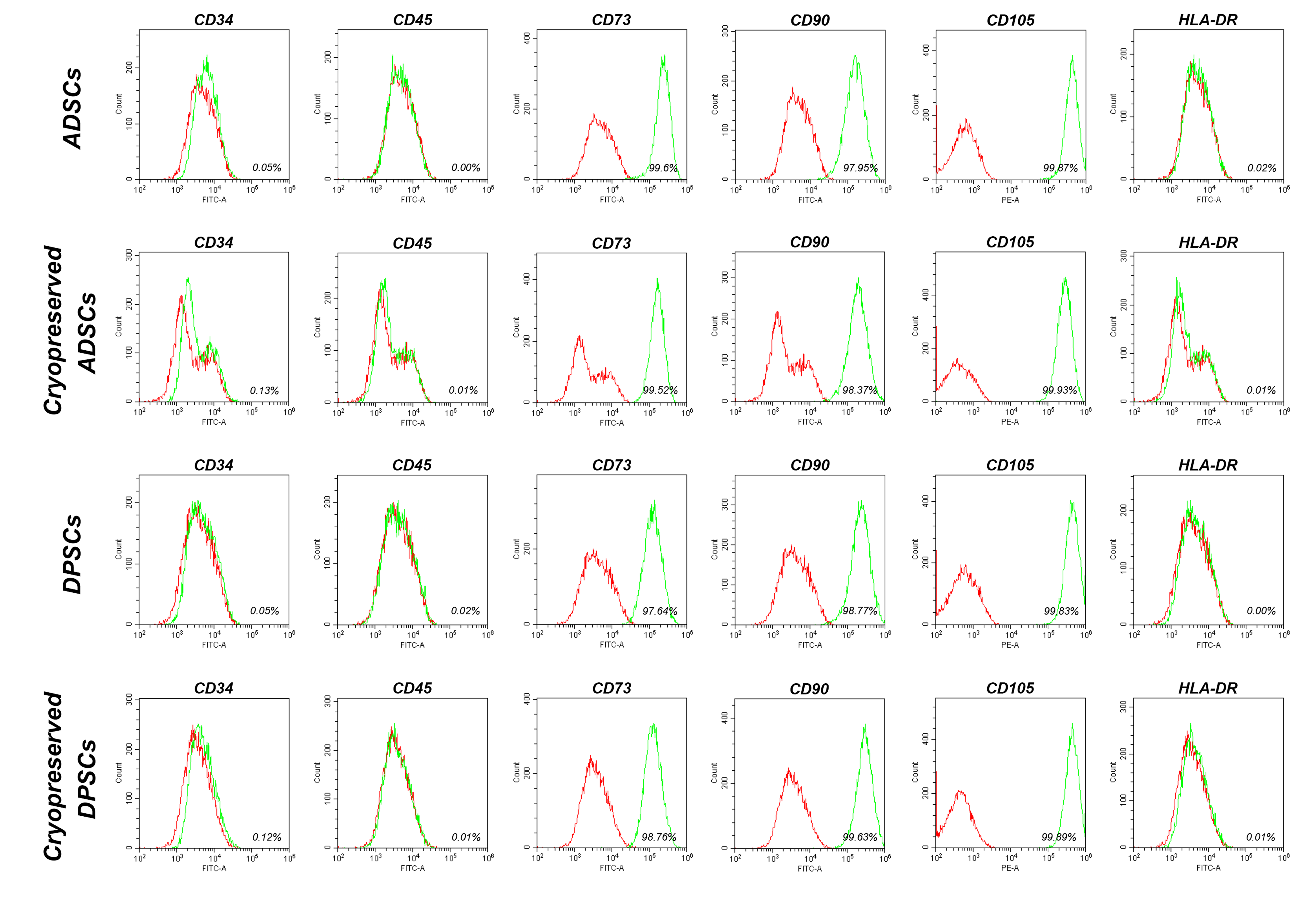

Supplement: Supplementary Figure 1 — Characterization of surface marker profiles for ADSCs and DPSCs prior to and after cryopreservation. The flow cytometry results revealed that the ADSCs and DPSCs were always positive for CD73, CD90, and CD105, but negative for CD34, CD45, and HLA-DR. [file Image_1.TIF]

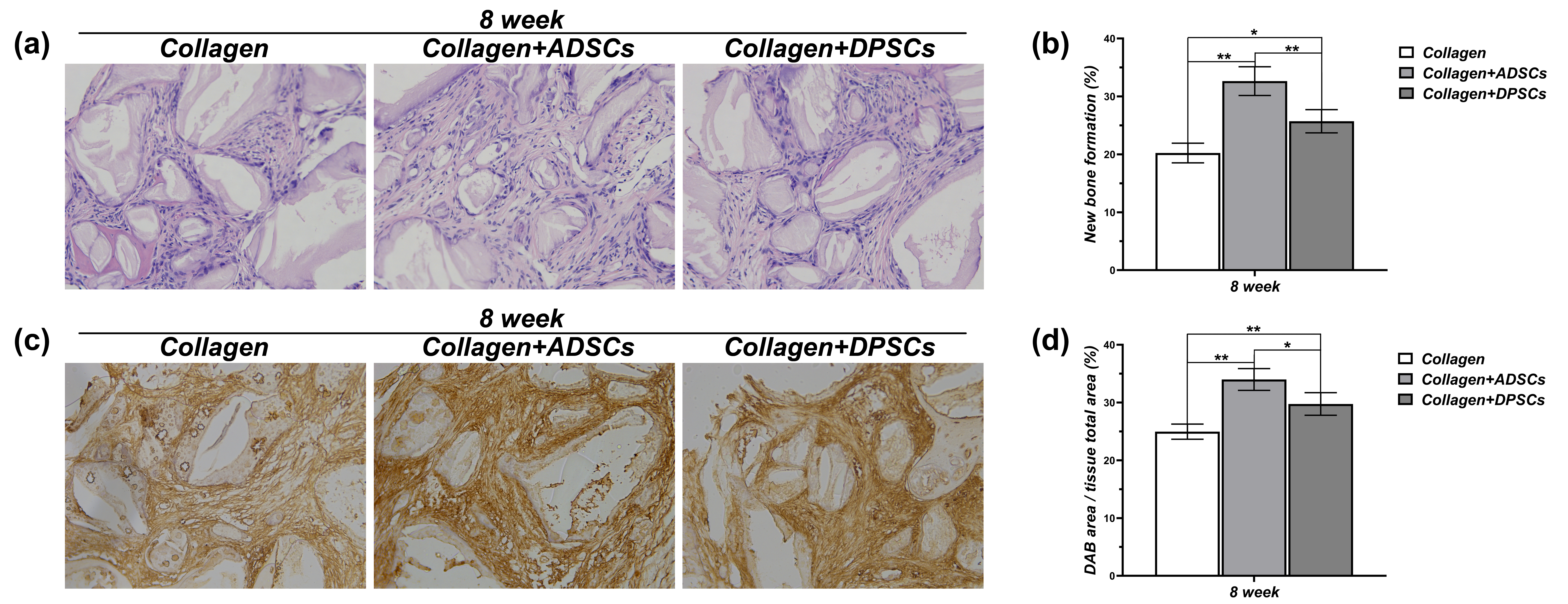

Supplement: Supplementary Figure 2 — Calvarial bone repair at 8 weeks in Control, Bio-Oss Collagen, Bio-Oss Collagen + ADSCs and Bio-Oss Collagen + DPSCs groups. (a) Hematoxylin and eosin (H&E) stained images. (b) New bone area percentages in the defect region as assessed by histomorphometric analyses. (c) Immunohistochemical staining specific for osteocalcin (OCN). (d) OCN immunostaining percentages as assessed by DAB total area/tissue total area analyses. Data are presented as the mean ± standard deviation, ∗P < 0.05, ∗∗P < 0.01. [file Image_2.TIF]
